# Candida dubliniensis Fungemia Leading to Infective Endocarditis and Septic Pulmonary Emboli

**DOI:** 10.7759/cureus.39031

**Published:** 2023-05-15

**Authors:** Abtin Jafroodifar, Ryan Thibodeau, Ernest Scalzetti

**Affiliations:** 1 Radiology, State University of New York Upstate Medical University, Syracuse, USA; 2 Radiology, Albany Medical Center, Albany, USA

**Keywords:** candida dubliniensis, computed tomography (ct) imaging, fungemia, broken needle fragments, infective endocarditis

## Abstract

Illicit drugs, especially those injected intravenously, are becoming increasingly more common worldwide. Individuals who use intravenous drugs often reuse or share needles which predisposes them to life-threatening infections. We present the case of a patient who was injecting intravenous drugs into her internal jugular vein, which eventually led to acutely worsening sepsis secondary to fungal infective endocarditis and bilateral septic pulmonary emboli. Transthoracic echocardiogram demonstrated multilobulated and spherical vegetations on the tricuspid and mitral valves, respectively. On computed tomography of the thorax, numerous cavitary lesions and ground-glass opacities were present in both lungs. Multiple hyperdense, linear structures consistent with broken needles were seen on chest radiography. It is important for radiologists to recognize the possibility of broken needles in patients with a history of intravenous drug use as astute recognition of broken needles may lead to better source control and improved outcomes.

## Introduction

Illicit drug use is becoming increasingly more common worldwide with approximately 11.7% of individuals 12 years and older having used some illicit drug in their lifetime [1]. Many illicit drugs are injected intravenously, raising the concern for both acute and chronic changes associated with continuous injections, as well as the potential complications associated with using previously used needles. One serious potential complication of intravenous drug use (IVDU) is systemic infections with a high mortality and morbidity rate through either direct inoculation or retained foreign objects following an injection, such as a broken needle. We report a case in which a young female presented with an acutely worsening systemic fungal infection secondary to IVDU, as evidenced by multiple broken needle shafts in her neck.

## Case presentation

A 29-year-old female with a past medical history of Ehlers-Danlos disease, treated chronic hepatitis C, and a history of intravenous heroin use presented to the emergency department with a persistent cough and myalgias for 1.5 weeks. The patient denied any recent IVDU and stated she has been on methadone maintenance therapy since late 2013. She denied any fevers and chest pain but reported dyspnea. Vitals signs demonstrated tachypnea and tachycardia consistent with a systemic inflammatory response. The physical examination was otherwise normal.

**Figure 1:**
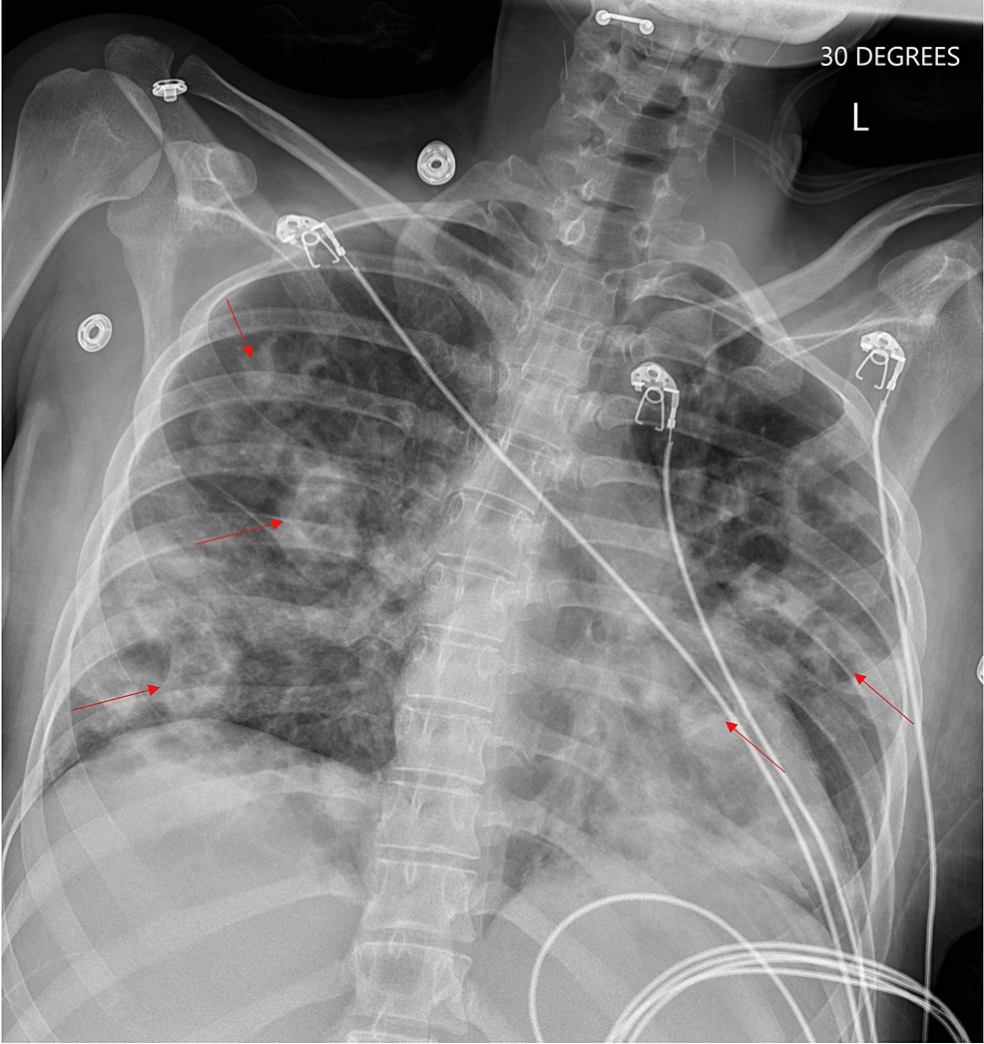
Portable 30 degrees upright anteroposterior chest radiograph demonstrates numerous bilateral lung opacities with central areas of lucency (red arrows). There is no obvious pleural effusion. The cardiac silhouette appears enlarged.

Laboratory values soon after presentation revealed leukocytosis with elevated absolute neutrophil count, evidence of acute kidney injury with elevated creatinine, lactic acidosis, and elevated C-reactive protein. Urine toxicology was positive for amphetamines, cocaine, fentanyl, methadone, opiates, and oxycodone. Blood cultures were positive for methicillin-resistant *Staphylococcus aureus* (MRSA) and the patient was started on vancomycin. Chest radiography demonstrated multiple patchy opacities with internal lucency in the middle and lower zones, likely secondary to multifocal necrotizing pneumonia or septic emboli (Figure 1). A transthoracic echocardiogram revealed a large (2.3 × 1.7 cm), multilobulated vegetation on the tricuspid valve associated with moderate tricuspid valve regurgitation. A small (0.8 × 0.6 cm), spherical vegetation was noted on the mitral valve.

**Figure 2:**
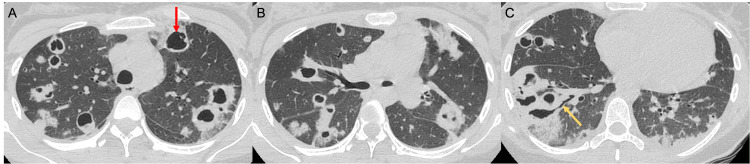
Axial CT images of the thorax with lung algorithm demonstrate numerous cavitary lesions throughout all lobes of the lungs. The largest lesion measures 3.5 cm in the greatest dimension in the anterior left upper lobe (red arrow in A). There are scattered areas of bronchiectasis (yellow arrow in C).

**Figure 3:**
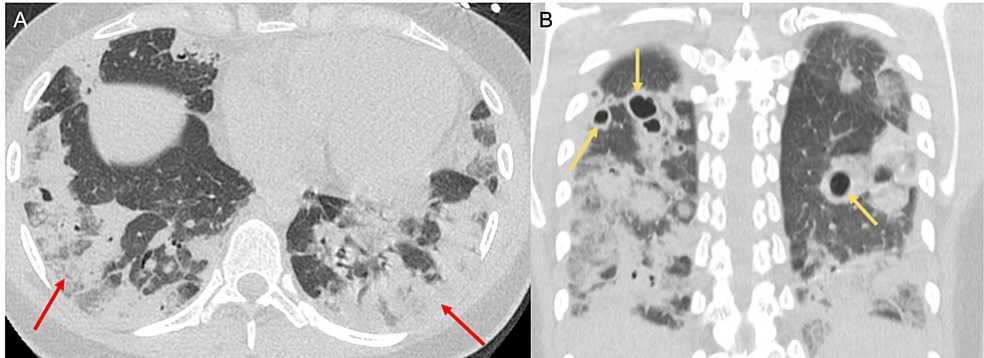
Axial (A) and coronal (B) CT images of the chest in the lung window demonstrate dependent ground-glass opacities involving bilateral lower lobes (red arrows in A). Cavitary lesions are present throughout the lungs (yellow arrows in B).

A follow-up computed tomography (CT) scan of the thorax demonstrated numerous thick-walled irregular cavitary lesions throughout both lungs, with the largest cavitary lesion measuring approximately 3.5 cm in greatest diameter (Figure 2). There were ground-glass opacities in the dependent portions of the lower lobes bilaterally (Figure 3). No evidence of pleural space disease or cardiovascular involvement was noted.

**Figure 4:**
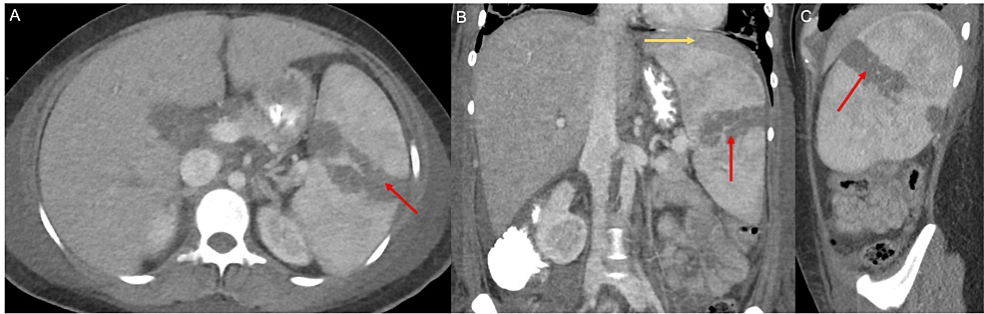
Axial (A), coronal (B), and sagittal (C) CT images of the abdomen with intravenous contrast demonstrate a 6.9 cm linear hypodensity in the splenic parenchyma (red arrow in A, B, and C) with a small amount of perisplenic fluid (yellow arrow in B), consistent with a grade 3 laceration.

CT of the head and spine demonstrated no septic emboli and epidural abscesses, respectively. CT of the abdomen and pelvis demonstrated an incidental trans-splenic laceration measuring 6.9 cm, concerning for a grade 3 splenic laceration (Figure 4). The surgical team determined no need for acute intervention after clinical and radiological review.

**Figure 5:**
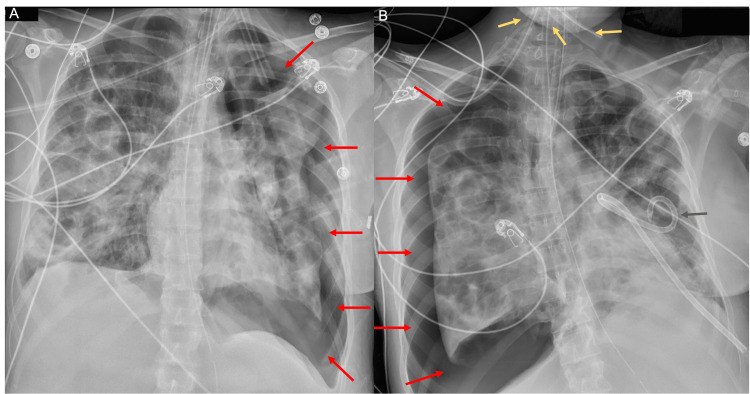
Portable semi-erect anterioposterior radiograph of the chest demonstrates a left pneumothorax (delineated by red arrows in A) without mediastinal shift. A subsequent radiograph of the chest demonstrates a large right pneumothorax (delineated by red arrows in B) with a possible right-to-left mediastinal shift. Thin, linear, metallic foreign bodies are noted involving the base of the neck (yellow arrows in B). A left-sided chest tube is present (dark gray arrow in B).

The patient became acutely hemodynamically unstable. She was intubated, sedated, paralyzed, and started on a vasopressin and norepinephrine drip. The patient’s clinical status once again deteriorated with oxygen desaturations to 70-72%, requiring bag-mask ventilation with the positive end-expiratory pressure valve turned to 20 cm H_2_O. Chest radiography obtained immediately after the incident demonstrated a new, large left pneumothorax extending from the apex of the left hemidiaphragm without evidence of mediastinal deviation, most likely secondary to a ruptured cavitary lesion (Figure 5, Panel A). On a detailed physical examination of the soft tissue of the neck, signs of fibrotic, chronic-appearing subcutaneous injection sites were present.

Given the development of pneumothorax, a chest thoracostomy tube was placed on the left, but the patient’s oxygenation status acutely worsened several hours later. Chest radiography demonstrated a right-sided pneumothorax with partial right pulmonary collapse, depression of the right hemidiaphragm, and possible mild mediastinal deviation from the right to the left (Figure 5, Panel B). There were multiple hyperdense, thin, linear objects located in the patient’s neck subcutaneously, which were favored to represent broken needle shafts (Figure 5B). A right-sided chest thoracostomy tube was placed. However, given continuous air leaks in the bilateral chest thoracostomy tubes and worsening right pneumothorax, a third right-sided chest thoracostomy tube was placed.

**Figure 6:**
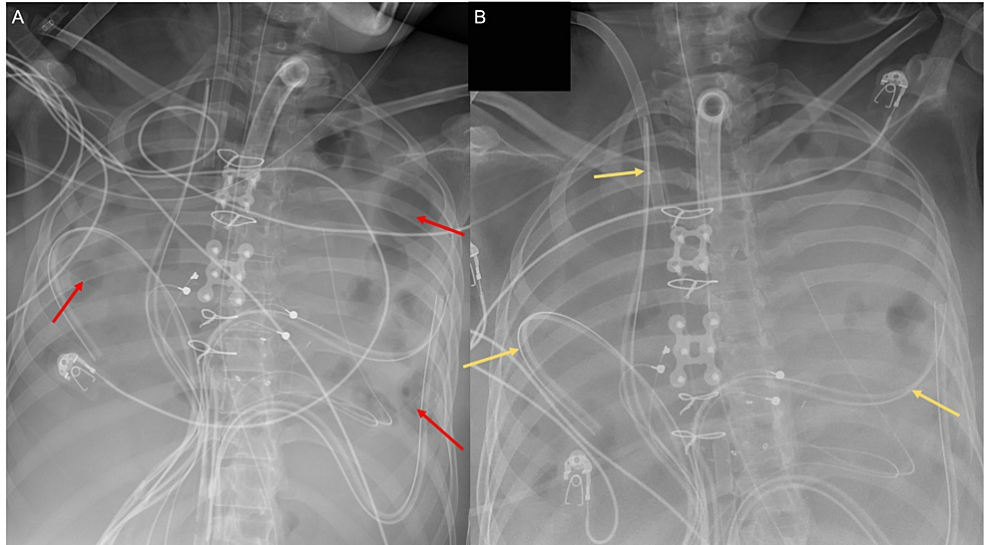
Portable semi-erect anterioposterior radiographs of the chest (A, B) demonstrate bilateral large pleural effusions with areas of loculations. Focal areas of aeration are present (red arrows in A). The patient is status post-sternotomy with sternal wires and closure devices in place. Numerous thoracic drains are in place (yellow arrows in B).

The cardiothoracic team was consulted due to the enlarging vegetations and worsening valvular regurgitation. The patient underwent surgery for mitral valve repair, bioprosthetic tricuspid valve replacement, atrial and ventricular epicardial lead placement, and initiation of venovenous extracorporeal membrane oxygenation (VV ECMO). Cultures from the mitral and tricuspid valves and pleural fluid were positive for MRSA. Although the patient was no longer bacteremic and her oxygen saturations began to improve, she had worsening bilateral pleural effusions with areas of loculation (Figure 6). A follow-up CT of the thorax demonstrated a right hydropneumothorax with areas of loculation and large areas of atelectasis of the right middle and lower lobes. The left upper lobe was almost entirely collapsed. There was a re-demonstration of multiple large, bilateral cavitations with variable wall thickness and internal soft tissue components. Culture from the pleural fluid and blood grew *Candida dubliniensis*.

**Figure 7:**
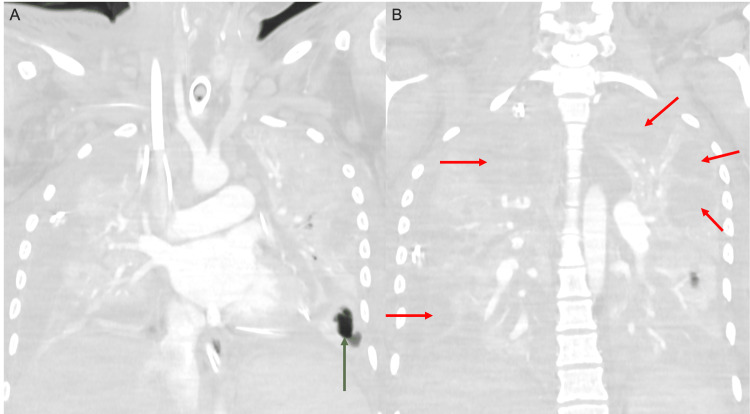
Coronal contrast-enhanced CT images of the chest in the lung window (A, B) demonstrate near-complete opacification of all lobes of the lungs bilaterally, with a small focal area of aeration involving the left lower lobe (green arrow in A). Multiple cystic collections are also present throughout the lungs (red arrows in B).

Given the persistence of air leaks in the bilateral chest thoracostomy tubes, the possibility of multiple broncho/alveolar-pleural leaks was suggested. The patient received a tracheostomy, was weaned off positive-pressure ventilation, and her respiratory requirements were supported with VV ECMO to allow the bilateral lungs and potential fistulae to heal. Over the course of several days, there was gradual and complete opacification of each hemithorax, consistent with acute respiratory distress syndrome secondary to lung damage. A CT of the thorax demonstrated near-complete atelectasis and consolidation of the bilateral lungs with multiple cystic air-fluid structures throughout the lung parenchyma, particularly in the peripheral locations (Figure 7). There were accompanying moderate-to-large bilateral pleural effusions.

The patient continued to deteriorate with laboratory values indicating worsening sepsis, likely with the lungs as the probable source of infection. She was referred to a nearby lung transplant center, but she was not a candidate given her persistent critical illness and infection, history of Ehlers-Danlos disease, history of hepatitis C, and history of IVDU. The palliative care team met with the family, who signed a do-not-resuscitate order. Within several hours, the patient became acutely tachycardic and hypotensive and began desaturating further. The family decided to pursue comfort care and the patient died several hours later.

## Discussion

Illicit drug use is a public health problem that affects all demographics. Approximately 269 million individuals aged 16-64 years used drugs in 2018, of which over 66 million individuals were from North America [1]. Globally, almost 9 million individuals (0.22% of the total population) inject intravenous drugs [2]. Due to unsterilized injection sites, sharing and repetitive use of needles, drug-dissolving agents, and poor socioeconomic living conditions, individuals using intravenous drugs are at high risk of bacterial infections. Sharing and repetitive use of needles are reported in approximately 50% of IVDUs worldwide [3].

Broken needles, most commonly when the needle shaft separates from the hub, are relatively common in IVDU [4]. Norfolk et al. found that 20% of IVDU cases experienced a broken needle at some point during their lifetime [5]. The main concern in this population is the sequelae of bacteremia, especially given the increased propensity of infection in individuals reusing or sharing needles. Infective sequelae include infective endocarditis (IE), mycotic aneurysm formation, septic thrombophlebitis, and septic emboli [6].

Using our case as an example, IE is a severe complication of bacteremia secondary to IVDU. Despite the increasing incidence of IVDU and iatrogenic causes of IE, left-sided IE is far more common (90-95%) than right-sided IE (5-10%) owing to several physiological and pathological differences between the right- and left-sided valves (oxygenation status, pressure gradient, turbulence, properties and vascularity of the endothelium, and prevalence of congenital malformations) [7]. IE in IVDU is estimated to occur 50-100-fold more often compared to those without IVDU which is concerning given the high one-year mortality rate (30%) [8]. IVDU heavily predisposes patients to right-sided IE, with involvement of the tricuspid valve (50-70%) being the most common site [9]. *Staphylococcus aureus* is the most common causative microbe (60-70%) in IE cases secondary to IVDU, though, notably, the second most common cause is MRSA (26%) [9].

Regardless of the microbial etiology, IE is well demonstrated with transthoracic or transesophageal echocardiography as an isoechoic, independently moving mass that is often associated with valvular regurgitation. In some cases, the diagnosis may be made using a multidetector CT of the thorax as a nodular and/or linear low-to-intermediate attenuated lesion of variable sizes along the valve, endocardium, or prosthesis. Migration of vegetation may result in septic emboli, with larger (>10 mm), mobile vegetations at a high risk of embolization. On contrast-enhanced studies, a filling defect may be detected [6,10]. Fungal vegetations tend to be bulkier than bacterial vegetations. Depending on the disease duration, disease burden, size, location, and characteristics of the vegetation itself, fungal vegetations may be difficult to differentiate from normal tissue or benign, nonspecific degenerative changes [11]. For the detection of valvular or perivalvular abscesses and pseudoaneurysms, recent data suggest that ECG-gated cardiac CT with thin-section reconstruction is at least equivalent to the current gold standard of transesophageal echocardiography. Combining the two modalities further improves sensitivity. However, transesophageal echocardiography is superior to cardiac CT for depicting small vegetations (<10 mm), perforations of valvular leaflets, and perivalvular leaks. In patients in whom transesophageal echocardiography is nondiagnostic or contraindicated, cardiac CT may be beneficial as an adjunctive imaging modality [10].

Although classically thought of as originating from IE, septic emboli may arise from any infectious source that leads to bacteremia, such as septic arthritis, skin and soft tissue infection, mouth or gingival disease, deep venous thrombosis, or foreign objects (i.e., broken needle) [12]. The most common foci of bacteremia include the urinary, lower respiratory, and gastrointestinal tracts [13]. The distribution of foci varies depending on where it was acquired, as well as the isolated microorganism. Community-acquired bacteremia is often caused by infections of the urinary tract or lower respiratory tract while healthcare-associated and nosocomial bacteremia is often from catheter-related infections [14]. Although septic emboli may distribute to any organ, locations often affected include the brain, lungs, kidney, liver, spleen, bone, skin, and eyes.

Imaging findings of septic emboli are dependent on the affected organ. Although nonspecific, chest radiography may demonstrate unilateral or bilateral, poorly marginated lung nodules often associated with cavitary changes and/or peripheral, lower lobe predominant opacities. These nodules may vary greatly in size depending on the disease burden and duration. Empyema may also be seen [15]. Similar to chest radiography, CT of the thorax often demonstrates nodular lesions. In fact, a study by Goswami et al. revealed that among the septic embolic lesions in 41 patients, all patients had nodular lesions while only 54% of patients had non-nodular lesions. A significant proportion (71%) of patients had cavitary lesions and associated pleural effusions (51%). Approximately 10% of patients had ground-glass changes. Due to the *embolic shower*, the vast majority of patients had greater than two lesions (93%), with as many as 36% of patients having more than 10 lesions [12]. CT may also demonstrate subpleural, wedge-shaped heterogenous and hyperattenuated areas with rim-like peripheral enhancement. Oftentimes, a vessel leading directly to the nodule (*feeding vessel*) may be seen [15].

Though a much rarer than bacterial infection, a fungal agent may lead to distributive septic emboli, with predominance in the lungs. Septic emboli may be the first and only symptom of fungal IE. Most cases of fungal IE are identified postmortem as it is difficult to isolate with cultures and has a high mortality ratio (50%) [16]. Among all cases of IE, fungal IE comprises anywhere from 1% to 10% of etiologies in which approximately 10% of agents originated from a bloodstream infection, with *Candida albicans *(46%) found most frequently [16]. *Candida dubliniensis* is far less common but has been linked to infected intracardiac devices [17]. *Candida dubliniensis* was first identified in 1995 as a phenotypically and genetically different species than *Candida albicans *though it is frequently mistakenly identified as *Candida albicans* [18]. *Candida dubliniensis* is often associated with IVDU [19].

While the majority of patients inject intravenous drugs in the distal upper extremity (antecubital fossa, hand, and forearm), patients may begin to inject in other areas of the body (neck, groin, finger, and shoulder) once the frequently accessed vessels begin to undergo sclerosis and occlude blood flow [20]. Despite the direct inoculation of contaminated skin, it is important to consider other sources of infection such as a retained foreign object (i.e., broken needle). Our patient injected intravenous drugs into her internal jugular vein, as evidenced by the multiple broken needles in her subcutaneous neck tissue. Injection into the internal jugular vein provides direct venous access to the right side of the heart and subsequently the bilateral lungs and left side of the heart.

Our patient is an unfortunate example of a severe microbial infection that may result from IVDU. Typically, in the setting of IE, the primary insult is most likely caused by *Staphylococcus aureus* followed by an opportunistic superinfection by a fungal species, specifically *Candida dubliniesis* in the case presented. Although the patient’s initial presentation was most likely secondary to bacteremia, the presence of fungemia complicated the patient’s clinical course and outcome. Additionally, the presence of broken needles throughout the soft tissues of the neck complicated the ability for source control and may have been harboring *Candida dubliniensis* as it is often linked to IVDU, as mentioned above [19]. However, it is inevitably unknown whether the fungemia resulted from direct inoculation or the chronically retained broken needles themselves. Nevertheless, radiologists should be cognizant of the possibility and frequency of broken needles in patients with a history of IVDU. Regardless of bacterial or fungal etiology, astute recognition of broken needles at common and uncommon injection sites may lead to better source control and improved outcomes.

## Conclusions

Retained broken needles are a common phenomenon in patients who use intravenous drugs. Whether through direct inoculation of a previously used or retained needle, patients are at high risk of bacterial and fungal infections. In particular, *Candida dubliniensis* is a fungal opportunistic pathogen that is often associated with intravenous drugs. Like bacterial infections, fungal bloodstream infections may result in IE and septic pulmonary emboli, which are associated with high mortality rates.

## References

[1] (2023). United Nations on Drugs and Crime. Prevalence-general. Published.

[2] (2023). United Nations on Drugs and Crime. People Who Inject Drugs. Published.

[3] Chu TX, Levy JA (2005). Injection drug use and HIV/AIDS transmission in China. Cell Res.

[4] Monroe EJ, Tailor TD, McNeeley MF, Lehnert BE (2012). Needle embolism in intravenous drug abuse. Radiol Case Rep.

[5] Norfolk GA, Gray SF (2003). Intravenous drug users and broken needles--a hidden risk?. Addiction.

[6] Hagan IG, Burney K (2007). Radiology of recreational drug abuse. Radiographics.

[7] Goud A, Abdelqader A, Dahagam C, Padmanabhan S (2015). Isolated pulmonic valve endocarditis presenting as neck pain. J Community Hosp Intern Med Perspect.

[8] Ternhag A, Cederström A, Törner A, Westling K (2013). A nationwide cohort study of mortality risk and long-term prognosis in infective endocarditis in Sweden. PLoS One.

[9] Jain V, Yang MH, Kovacicova-Lezcano G, Juhle LS, Bolger AF, Winston LG (2008). Infective endocarditis in an urban medical center: association of individual drugs with valvular involvement. J Infect.

[10] Saeedan MB, Wang TK, Cremer P (2021). Role of cardiac CT in infective endocarditis: current evidence, opportunities, and challenges. Radiol Cardiothorac Imaging.

[11] Horgan SJ, Mediratta A, Gillam LD (2020). Cardiovascular imaging in infective endocarditis: a multimodality approach. Circ Cardiovasc Imaging.

[12] Goswami U, Brenes JA, Punjabi GV, LeClaire MM, Williams DN (2014). Associations and outcomes of septic pulmonary embolism. Open Respir Med J.

[13] Uslan DZ, Crane SJ, Steckelberg JM, Cockerill FR 3rd, St Sauver JL, Wilson WR, Baddour LM (2007). Age- and sex-associated trends in bloodstream infection: a population-based study in Olmsted County, Minnesota. Arch Intern Med.

[14] Rodríguez-Baño J, López-Prieto MD, Portillo MM (2010). Epidemiology and clinical features of community-acquired, healthcare-associated and nosocomial bloodstream infections in tertiary-care and community hospitals. Clin Microbiol Infect.

[15] Kuhlman JE, Fishman EK, Teigen C (1990). Pulmonary septic emboli: diagnosis with CT. Radiology.

[16] Pierrotti LC, Baddour LM (2002). Fungal endocarditis, 1995-2000. Chest.

[17] Merza N, Lung J, Bainum TB, Mohammedzein A, James S, Saadaldin M, Naguib T (2020). First reported case of Candida dubliniensis endocarditis related to implantable cardioverter-defibrillator. Case Rep Cardiol.

[18] Sullivan DJ, Westerneng TJ, Haynes KA, Bennett DE, Coleman DC (1995). Candida dubliniensis sp. nov.: phenotypic and molecular characterization of a novel species associated with oral candidosis in HIV-infected individuals. Microbiology (Reading).

[19] Meiller TF, Jabra-Rizk MA, Baqui Aa, Kelley JI, Meeks VI, Merz WG, Falkler WA (1999). Oral Candida dubliniensis as a clinically important species in HIV-seropositive patients in the United States. Oral Surg Oral Med Oral Pathol Oral Radiol Endod.

[20] Pong TM, Oflazoglu K, Helliwell LA, Chen NC, Eberlin KR (2019). Intravenous drug use-related complications of the hand and upper extremity. Plast Reconstr Surg Glob Open.

